# *In Vitro* Virucidal and Virustatic Properties of the Crude Extract of *Cynodon dactylon* against Porcine Reproductive and Respiratory Syndrome Virus 

**DOI:** 10.1155/2014/947589

**Published:** 2014-03-09

**Authors:** Kidsadagon Pringproa, Oapkun Khonghiran, Suchaya Kunanoppadol, Teerapong Potha, Phongsakorn Chuammitri

**Affiliations:** ^1^Department of Veterinary Biosciences and Veterinary Public Health, Faculty of Veterinary Medicine, Chiang Mai University, Chiang Mai 50100, Thailand; ^2^Central Veterinary Diagnostic Laboratory, Faculty of Veterinary Medicine, Chiang Mai University, Chiang Mai 50100, Thailand

## Abstract

The *in vitro* virustatic and virucidal tests of the crude extract of *Cynodon dactylon* against infection with porcine reproductive and respiratory syndrome virus (PRRSV), a cause of major devastating pig disease, were described. Crude extract of *C. dactylon* was prepared for cytotoxicity on tissue-culture cells that were used to measure virustatic and virucidal activities against PRRSV. Crude extract of *C. dactylon* at 0.78 mg/mL showed no cytotoxicity on the cell line, and at that concentration significantly inhibited replication of PRRSV as early as 24 hours post infection (hpi). *C. dactylon* also inactivated PRRSV as determined by immunoperoxidase monolayer assay (IPMA) compared to the control experiments. In summary, the present study may be among the earliest studies to describe virustatic and virucidal activities of *C. dactylon* crude extract against PRRSV *in vitro*. Extracts of *C. dactylon* may be useful for PRRSV control and prevention on pig farms.

## 1. Introduction 

Porcine reproductive and respiratory syndrome (PRRS) is an important, viral infectious disease that affects the swine industry worldwide [1, 2]. PRRS virus causes reproductive failure in breeding pigs, increases mortality in young pigs, and exerts severe respiratory disease and poor growth performance in all ages of infected pigs [3–5]. Although PRRS vaccines have been introduced in pig farms, their effectiveness and safety remain unclear [6, 7]. As a result, recurrent PRRS outbreaks on pig farms are a serious problem. Thus pharmacologic agents that would inhibit PRRSV replication are a central goal in research [8–11].

*Cynodon dactylon*, also known as Bermuda grass, is a common perennial native grass that grows naturally and ornamentally in the tropical and warm temperate regions [12, 13]. Belonging to the  family Poaceae,* C*.* dactylon* is claimed to have antimicrobial, antidiabetic, antihyperlipidemia, anti-inflammatory, and antiemetic activities [12, 14, 15]. In addition, the extract is also diuretic and traditionally known for treatment of urinary tract infections and uroliths [16, 17].* C*.* dactylon* has recently been demonstrated to exert a strong protective effect on right-sided heart failure in rats [12]. Furthermore, its extract has been described to have potential antiviral activity against white spot syndrome virus (WSSV) of shrimp both* in vitro* and* in vivo* [18]. The aims of this study were to explore the virustatic and virucidal activities of* C*.* dactylon* extract against PRRSV infection* in vitro*, toward a potentially feasible application of an inexpensive natural plant as an alternative antiviral agent in veterinary medicine.

## 2. Materials and Methods

### 2.1. Plants and Preparation of the Extract

Naturally growing plants of* C. dactylon* were collected from Sribuabarn Subdistrict, Muang District of Lumphun Province, Thailand, from June to July 2011. Specimens were botanically confirmed for identity by the Department of Biology, Faculty of Science, Chiang Mai University, Chiang Mai, Thailand. Crude extract of dried* C. dactylon* (250 g) was obtained by macerated with 95% ethanol (1,000 mL) at room temperature (RT) for 24 hours. The extract solutions were filtered with Whatman No. 1, concentrated under a rotary evaporator (Buchi, Flawil, Switzerland), and finally lyophilized (Snijders Scientific, Tilburg, The Netherlands) to obtain a dried crude extract. This dried crude extract was resuspended with 1% dimethyl sulfoxide (DMSO, Bio Basic Inc., NY, USA), aliquoted to a final dilution of 100 mg/mL, and kept at −20°C for use as a study reagent.

### 2.2. Cell Culture and Viruses

MARC-145 tissue culture cells were grown in Dulbecco's Modified Eagle Medium (DMEM; PAA Laboratories GmbH., Pasching, Austria) supplemented with 10% fetal bovine serum (FBS; PAA Laboratories GmbH., Pasching, Austria) and 1% Pen/Strep at standard culture conditions (37°C, 5% CO_2_). The media were changed every two to three days. The US genotype of PRRSV (kindly provided by Professor R. Thanawongnuwech, Faculty of Veterinary Sciences, Chulalongkorn University, Bangkok, Thailand) was propagated in the MARC-145 cells, and the virus titer was quantified by the immunoperoxidase monolayer assay (IPMA), as previously described [19]. The final titer was 10^4.5^ 50% tissue culture infectious dose (TCID_50_)/mL.

### 2.3. Cytotoxicity Testing

Cytotoxicity of the study reagent on the MARC-145 cells was studied by both staining with crystal violet and MTT assay (Sigma-Aldrich Co., St. Louis, MO, USA) as previously described [11]. In brief, MARC-145 cells were incubated in 96-well microtiter plates (Nunc, Roskilde, Denmark) for 24 hours at standard culture conditions. Two fold serial dilutions of* C. dactylon* study reagent were mixed in DMEM/10% FBS and added in triplicate to the semiconfluent cells. Medium without study reagent served as a control. After 72 hours, the cells were either fixed or stained with crystal violet, or their viability was assessed by MTT test. The highest concentration of* C. dactylon* that did not kill the cells was chosen and used for further studies.

### 2.4. Virustatic Test

MARC-145 cells grown in 96-well microtiter plates were infected with PRRSV at a multiplicity of infection (MOI) of one, for one hour at 37°C. Then unbound viruses in supernatants were removed, and the cells were replaced with medium (DMEM/10% FBS) containing with 0.78 and 0.36 mg/mL of* C. dactylon* study reagent. The control was medium with DMSO added to 1% concentration, but without study reagent. The plates were incubated under standard culture conditions, and at time points 24, 48, and 72 hours post infection (hpi), their supernatants were collected and quantified for virus titer. Experiments were done in triplicate. Virus titers were analyzed by the presence of mean and standard error as previously described [19].

### 2.5. Virucidal Test

The virucidal effect of* C. dactylon* study reagent toward PRRSV was evaluated as previously described [20]. Briefly,* C. dactylon* study reagent at a concentration of 0.78 mg/mL was mixed with 10^4.5^  TCID_50_/mL of PRRSV in a ratio of 1 : 1 and incubated for one hour at RT. DMSO (1%) containing DMEM mixed with PRRSV served as a positive control. After one hour, the mixtures of virus and study reagent, or virus and DMEM control, were inoculated onto a monolayer of MARC-145 cells and incubated for one hour at 37°C. The unbound viruses were then discarded, and the old medium was replaced with fresh DMEM containing 10% FBS. After three days* in vitro*, the viability of PRRSV after inactivation with study reagent was determined. Data were analyzed as described above.

### 2.6. Statistical Analysis

Statistical analyses were performed with GraphPad Prism 5 (Graph Pad Inc., La Jolla, CA, USA). One-way analysis of variance (ANOVA) with Tukey's* post hoc* test of means was used for multiple comparisons among treatment groups. Student's* t*-test was used to compare the means of two independent groups. Statistical significance was designated as *P* ≤ 0.05.

## 3. Results/Discussion

Because of public health concern for drug residues in pork and poultry, alternative compounds to control bacterial and viral infections in pigs are sought [21–23]. One such pathogen is the porcine reproductive and respiratory syndrome virus (PRRSV), a major, devastating viral disease in pigs, for which vaccine strategies have failed to control outbreaks [6, 7], and only a few reports have described the use of medicinal compounds against it [8, 11, 24]. The present study aimed to evaluate the antiviral activity of* C. dactylon *against PRRSV* in vitro*.

**Figure 1:**
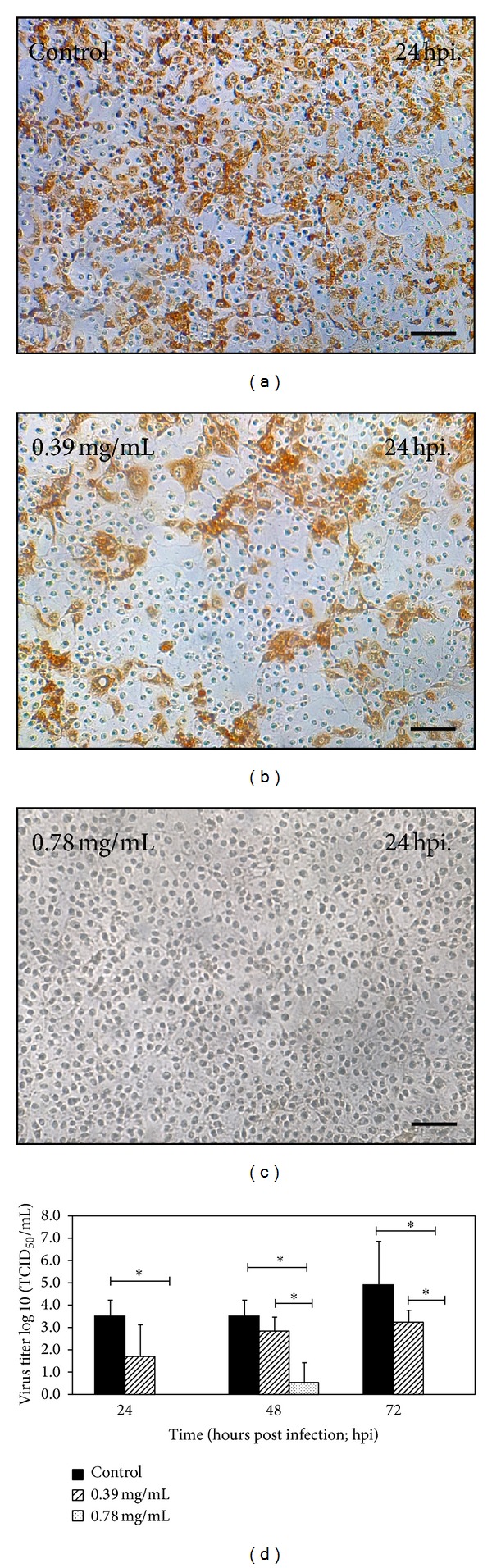
Virustatic activity of* C. dactylon* study reagent on replication of porcine reproductive and respiratory syndrome virus (PRRSV). MARC-145 cells were infected with PRRSV at a multiplicity of infection of 1. At various time points after infection, PRRSV in supernatants were quantified for virus titer by immunoperoxidase monolayer assay. In control group, viruses in supernatants at 24 hpi were infected and replicated in the MARC-145 cells (a), while little or no PRRSV were observed in groups of study reagent (b, c). At the concentration of 0.78 mg/mL, virus could only be observed in supernatants of 48 hpi suggesting that PRRSV growth was interrupted by* C. dactylon*. Representative data are mean and standard error. Asterisks indicate statistical significance (*P*  value ≤ 0.05). Scale bar in (a, b, and c) *≈*180 *μ*m.

The cytotoxicity test of* C. dactylon* study reagent on MARC-145 cells revealed that, at the concentration of 0.78 mg/mL,* C. dactylon* had no cytotoxic effect on the tested cells. This concentration of* C. dactylon* study reagent was, therefore, chosen for further* in vitro* studies. The results of virustatic test showed that* C. dactylon* study reagent at 0.78 mg/mL had the ability to inhibit PRRSV replication as early as 24 hpi (Figure 1).** **In addition,* C. dactylon* study reagent at 0.39 mg/mL did not significantly diminish PRRSV replication compared to the control experiment (Figures 1(a), 1(b), and 1(d)). This finding suggested that only an appropriate concentration of* C. dactylon *could effectively inhibit PRRSV replication. We would like to stress the fact that this virustatic property of* C. dactylon* study reagent was not due to DMSO, since the control group was only DMSO containing medium. Furthermore, the virustatic activity of* C. dactylon *has been observed on shrimp against white spot syndrome virus (WSSV)* in vitro* and* in vivo* [18].

**Figure 2:**
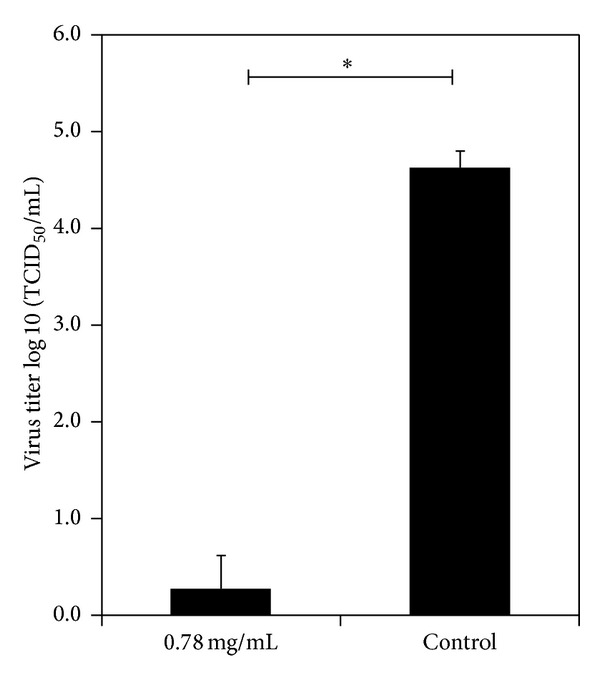
Virucidal activity on PRRSV of* C. dactylon* study reagent at a concentration of 0.78 mg/mL.* C. dactylon* study reagent was incubated with PRRSV for one hour at room temperature before inoculated onto a monolayer of MARC-145 cells. After three days* in vitro*, virus titer was quantified by IPMA.* C. dactylon* significantly inactivated PRRSV compared to the control group, while virus alone with DMEM remained infective on MARC-145 cells. Asterisks indicate statistical significance (*P*  value ≤ 0.05).

To determine whether the inhibitory activity of* C. dactylon *on PRRSV was due to the virucidal effect of the extract after new virions were released from infected cells, the virucidal test was conducted. After incubation of PRRSV with 0.78 mg/mL* C. dactylon *study reagent for one hour was then inoculated onto a monolayer of MARC-145 cells, and IPMA was done after three days in culture. The results indicated that study reagent at 0.78 mg/mL potentially inactivated PRRSV infectivity compared to the control experiment (Figure 2).

It has been shown that chemical constituents of ethanolic extraction of* C. dactylon* are composed of terpenoids, protein, and amino acids [25] and that terpenoids bear antiviral activity against several types of viruses, including herpes virus, Epstein-Barr virus, and Human metapneumovirus [26–28]. Though* C. dactylon* crude extract potentially inhibited replication and infection of PRRSV* in vitro*, the exact mechanism(s) underlying these findings need(s) to be investigated further.

## 4. Conclusion

This study indicates that crude extract of* C. dactylon* at the concentration of 0.78 mg/mL potentially inactivates PRRSV and inhibits replication of PRRSV* in vitro*. The results of this study may be useful for future application of medicinal plants to the PRRSV control and prevention in the pig farms.
